# Enhancing heart disease prediction with stacked ensemble and MCDM-based ranking: an optimized RST-ML approach

**DOI:** 10.3389/fdgth.2025.1609308

**Published:** 2025-06-19

**Authors:** T. Ashika, G. Hannah Grace

**Affiliations:** Department of Mathematics, School of Advanced Sciences, Vellore Institute of Technology Chennai, Chennai, India

**Keywords:** rough set theory, machine learning, correlation analysis, stacking classifier, multi-criteria decision-making, gridsearchCV

## Abstract

**Introduction:**

Cardiovascular disease (CVD) is a leading global cause of death, necessitating the development of accurate diagnostic models. This study presents an Optimized Rough Set Theory-Machine Learning (RST-ML) framework that integrates Multi-Criteria Decision-Making (MCDM) for effective heart disease (HD) prediction. By utilizing RST for feature selection, the framework minimizes dimensionality while retaining essential information.

**Methods:**

The framework employs RST to select relevant features, followed by the integration of nine ML classifiers into five stacked ensemble models through correlation analysis to enhance predictive accuracy and reduce overfitting. The Technique for Order of Preference by Similarity to Ideal Solution (TOPSIS) ranks the models, with weights assigned using the Mean Rank Error Correction (MEREC) method. Hyperparameter tuning for the top model, Stack-4, was conducted using GridSearchCV, identifying XGBoost (XG) as the most effective classifier. To assess scalability and generalization, the framework was evaluated using additional datasets, including chronic kidney disease (CKD), obesity levels, and breast cancer. Explainable AI (XAI) techniques were also applied to clarify feature importance and decision-making processes.

**Results:**

Stack-4 emerged as the highest-performing model, with XGBoost achieving the best predictive accuracy. The application of XAI techniques provided insights into the model's decision-making, highlighting key features influencing predictions.

**Discussion:**

The findings demonstrate the effectiveness of the RST-ML framework in improving HD prediction accuracy. The successful application to diverse datasets indicates strong scalability and generalization potential, making the framework a robust and scalable solution for timely diagnosis across various health conditions.

## 1 Introduction

CVD is a leading global cause of mortality, accounting for 17.9 million deaths annually, as estimated by the World Health Organization (WHO). CVD encompasses various heart and blood vessel conditions and is strongly associated with several modifiable risk factors, including stress, poor diet, physical inactivity, smoking, and excessive alcohol consumption. Factors such as obesity, hypertension, high cholesterol, and tobacco use further exacerbate its severity, emphasizing the need for early detection and intervention (1). Artificial Intelligence (AI) has revolutionized medical research, with ML playing a critical role in predictive modelling for clinical diseases (2).

AI and data mining techniques facilitate early CVD risk prediction by analysing large-scale behavioural and clinical data, identifying patterns associated with disease progression, and supporting timely preventive interventions. While diagnosis may involve imaging, predictive models using ML, support pre-symptomatic risk assessment, enabling timely intervention (3, 4). Traditional statistical methods, while effective under specific data distributions, often face limitations when applied to non-linear, high-dimensional clinical datasets. These challenges have led to increased adoption of ML and DL techniques to improve predictive performance in complex healthcare scenarios (5).

ML excels in handling large-scale medical data, enabling early-stage disease prediction and reducing preventable hospitalizations. Its application enhances healthcare policies, disease prevention, and medical decision-making. Many studies have explored ML techniques for cardiac disease classification (6, 7). ML based computer aided decision support systems enhance diagnostic accuracy and optimize treatment strategies. However, challenges persist in feature selection and model efficiency, as medical datasets often contain redundant and inconsistent information, affecting predictive performance (8). Selecting the most relevant features is crucial for enhancing model accuracy, reducing overfitting, and improving interpretability (9).

Feature Selection methods eliminate redundant data while retaining critical features, improving classification accuracy. RST, introduced by Pawlak, is a powerful mathematical tool for handling uncertainty and incomplete data. It identifies minimal reduct sets while preserving classification accuracy, making it highly suitable for feature selection in ML models (10, 11). MCDM techniques aid in optimizing complex decision processes by distinguishing the best and worst alternatives based on multiple criteria. Decision-making frameworks, such as Fuzzy Set Theory (FST) and RST, effectively handle uncertainty and enhance classification accuracy (12, 13).

Despite advances in ML-based HD prediction, challenges remain in optimizing accuracy and minimizing model complexity. This study addresses these challenges by integrating RST with ML and employing an MCDM-based ranking system to enhance model performance and interpretability. RST with Johnson's algorithm reduces dimensionality by selecting the most informative features, improving both accuracy and interpretability; nine ML classifiers are evaluated and combined into five stacked ensembles through correlation analysis; and MCDM techniques, specifically TOPSIS with MEREC-based weighting, rank the stacked models to identify the best-performing approach, which is further optimized using GridSearchCV. Unlike conventional models that rely on individual classifiers or simple ensembles, this framework enhances accuracy, minimizes overfitting, and improves explainability through Shapley Additive exPlanations (SHAP) and Local Interpretable Model-agnostic Explanations (LIME). By integrating RST, diverse ML classifiers, and MCDM-based ranking, this study addresses key challenges in HD prediction and presents a scalable, robust framework with strong potential for clinical application in early disease detection. The key contributions of the study include,

• Employed RST to effectively eliminate redundant features and retain only the most relevant attributes, thereby enhancing the model's predictive accuracy while minimizing unnecessary complexity. This approach significantly improves HD diagnosis by focusing on essential features.
• Utilized correlation analysis to identify diverse base learners, constructing effective stacked ensemble models. This ensures a robust and versatile prediction model that can handle varied patterns in HD data, leading to enhanced model performance.
• Applied the TOPSIS technique, combined with MEREC-based weighting, to systematically rank the performance of stacked models.
• Fine-tuned Stack-4 using GridSearchCV, ultimately identifying XG as the top-performing classifier. This fine-tuning process led to superior predictive accuracy and ensured the model's robustness in real-world applications.
• Integrated XAI methods such as SHAP and LIME to analyse and interpret feature importance. These techniques enhance the transparency of the model, making it more clinically interpretable and ensuring that healthcare professionals can trust and effectively use the predictive model for early HD diagnosis.

The remainder of the paper is organized as follows**:** Subsection 1.1 reviews related work, while 1.2 outlines the motivation and highlights the novelty of the proposed approach. Section 2 outlines the materials and methods used in the proposed work. Section 3 presents the research findings. Section 4 discusses the findings and limitations, and Section 5 concludes the study and outlines future work.

### 1.1 Literature review

Numerous ML methods have been developed recently for the diagnosis of cardiac disease. Furthermore, a variety of medical diagnostics, such as those in Radiology, Dermatology, Hematology, and Ophthalmology, employ AI. HD is the leading cause of death in India, with a study showing a 29.4% prevalence among adults 45 and older. Key risk factors include age, gender, residence, high cholesterol, diabetes, inactivity, and family history, underscoring the need for targeted health programs and early detection efforts (14). Additionally, effective management has significantly improved outcomes for hemophilia patients; however, as they age, they face increased risks of HD, highlighting the imperative for enhanced understanding and management of HD in this population (15).

HD are globally significant, impacting mortality rates and healthcare costs. Timely diagnosis through advanced predictive models like ANN, feature selection methods, and MCDM techniques can effectively reduce fatalities and treatment expenses. Efficient feature selection and model validation methods highlight promising approaches, offering potential advancements in disease prediction methodologies (16). By providing better risk prediction using AI-based data integration approaches, AI has greatly improved traditional risk assessment tools, such as the Thrombolysis in Myocardial Infarction and Global Registry of Acute Coronary Events scores (17). Decision making techniques, particularly AHP and hybrid approaches, have been prominently used in healthcare contexts to evaluate service quality, offering insights and recommendations for future research directions (18).

Wearable technology monitoring of biopotential signals is critical for HD tracking and early detection. Current developments in wearable biosensors have enhanced precision, reproducibility, and continuous monitoring, significantly reducing healthcare costs (19). Emerging technologies like the FlexiPulse sensor, designed for affordable and accurate HD monitoring, exemplify the advancements in wearable tech. This sensor achieves over 93% accuracy and uses ML to diagnose HD events with 98.7% accuracy (20). Innovative hybrid DL systems have demonstrated high accuracy in predicting HD, utilizing advanced preprocessing techniques and optimized feature extraction methods. For instance, a hybrid DL system achieved 99.12% accuracy across diverse benchmark datasets (21). Furthermore, ML techniques applied to heart failure prediction using advanced methods like XG have shown promising results, achieving accuracies up to 86.36%, highlighting their potential to enhance early mortality estimation and public health outcomes (22).

Furthermore, the analysis of HD has been improved by data mining techniques such as Recurrent Neural Network - Long Short-Term Memory models and genetic algorithms, which demonstrate progress if applied to clinical datasets (23). Novel ML methods, such as those optimized with N2Genetic optimizer, have shown superior accuracy and F1-scores in predicting coronary artery disease, emphasizing their utility in medical decision making. Use of ML in clinical practice extends from pre-clinical data processing to bedside applications, although challenges like validation in real-life settings and ethical considerations remain critical (24). Identification of those with a higher risk of early atherosclerosis and HD in adolescents with Major Depressive Disorder can facilitate individualized therapies and improve outcomes related to both cardiovascular and mental health (25). Using ML to precisely predict HD is essential for early detection and lowering death rates. The study compares several ML algorithms, identifying Random Forest (RF) as the most accurate, and underscores the potential of these technologies to improve healthcare outcomes (26). ML techniques applied to RNA-seq data identified significant genes linked to HD, enhancing early prediction capabilities. The study underscores the potential for these approaches in advancing personalized treatments and understanding disease heterogeneity (27).

In a large population-based study, ML survival models, developed from self-reported questionnaire data, slightly outperformed traditional classification methods in predicting hospitalization for ischemic HD and cardiovascular mortality, with logistic regression being the top-performing classification method. These models demonstrate promise as reliable tools for screening and identifying individuals at high risk (28). Recent healthcare research emphasizes the importance of early detection of HD, focusing on assessing the Right Ventricle through MRI image segmentation using ML and DL methods such as Fourier- Convolutional Neural Networks (F-CNN), Vanilla CNN, and ResNet. These techniques aim to improve accuracy in identifying the right ventricle abnormalities and enhancing decision making in HD treatment (29). Techniques like disease comorbidity network-based temporal DL framework, which integrates disease comorbidity networks and time-aware DL, enhance cardiovascular risk prediction, especially in patients with mental disorders (30).

Predictive models based on ML for assessing the risk of HD are designed to perform consistently across various demographic categories, such as gender and race. Techniques for mitigating bias are crucial to address systematic biases in health data collection and preprocessing, which can impact model performance on different demographic sub-cohorts. These models use electronic health records data and various ML algorithms to ensure fairness and accuracy in HD risk prediction across diverse populations (31). ML models, particularly RF with Synthetic Minority Oversampling Technique (SMOTE), achieve high accuracy (96.6%), sensitivity (90%), and specificity (100%) in early prediction of cardiac disease using UCI heart dataset (32). A novel multi-modal approach integrates ECG and PCG features to predict HDs. Genetic algorithms optimize feature subsets, and Support Vector Machine (SVM) classification achieves an AUC of 0.936 (33).

ML plays a vital role in healthcare by leveraging extensive datasets to predict HDs early, ensuring better detection and treatment outcomes. Fusion models combining outputs from multiple algorithms achieve high accuracy, reaching 93%–95% in binary classification and 72%–75% in multiclass classification of HD severity. This highlights their potential for early disease identification and risk assessment in healthcare settings (34). The “Sathvi” dataset, compiled from prevalent HD datasets, has boosted prediction accuracy using ML classifiers like CatBoost, achieving up to 98.11% accuracy through Cross-Validation (CV) (35). Various ML algorithms applied to clinical and pathological data, such as Gradient Boosting and XG, have shown that categorical features consistently outperform numerical and combined features, with SVM and AdaBoost achieving optimal performance in CVD prediction (36). Predicting the risks of CVD accurately during pregnancy is crucial for efficient treatment. A hybrid system that combines the Sugeno Fuzzy Inference System (S-FIS), Coefficient of Variation, and Fuzzy Analytic Hierarchy Process (F-AHP) demonstrated high accuracy, sensitivity, and precision, showing its potential in clinical settings for predicting CVD during pregnancy (37).

Unlike prior models that rely heavily on individual classifiers or lack interpretability, the proposed framework integrates RST for efficient feature reduction, stacked ensemble learning for enhanced robustness, and XAI techniques for improved transparency. This holistic approach not only boosts predictive performance but also supports clinical decision-making through interpretability. Additionally, Table 6 provides a detailed quantitative comparison of our model against existing state-of-the-art HD prediction methods, further justifying the proposed methodological choices.

### 1.2 Motivation and novelty

Traditional methods for predicting HD often face challenges in handling large volumes of data, high-dimensionality, and uncertainty in feature selection. The motivation for the proposed work arises from the need to improve prediction accuracy by addressing these challenges by combining RST for feature selection with ML techniques, and utilizing MCDM methods for model ranking. The goal is to develop a robust framework that enhances prediction accuracy by selecting the best-performing model through RST-based feature selection and MCDM ranking for HD prediction. The novelty of the proposed work lies in the integration of advanced methodologies to address persistent challenges in ML-based HD prediction:

1. The proposed work applies RST using Johnson's algorithm to reduce data dimensionality by identifying the most informative features. This process enhances model accuracy and interpretability by eliminating redundant attributes.
2. The framework evaluates nine diverse ML classifiers to determine the most effective algorithms for HD prediction. These classifiers are combined into five stacked ensembles through correlation analysis.
3. The incorporation of MCDM techniques, specifically TOPSIS with MEREC-based weighting, systematically ranks the stacked models. This approach identifies the best-performing stack for HD prediction, which is further optimized using GridSearchCV to determine the most effective ML model.

Unlike traditional models that rely on individual classifiers or simple ensembles, this integrated approach enhances prediction accuracy, minimizes overfitting, and offers clearer insights into feature importance through XAI techniques such as SHAP and LIME. By integrating RST, diverse ML classifiers, and MCDM-based ranking, the proposed work fills a critical research gap and presents a scalable, robust framework with strong potential for real-world clinical implementation in early HD detection.

## 2 Materials and methods

This section provides a comprehensive overview of the methodology employed in this study, detailing the dataset used, preprocessing steps, feature selection techniques, classifier selection, MCDM ranking, and hyperparameter tuning.

### 2.1 Proposed methodology

This section proposes a hybrid intelligent Optimized RST-ML approach with MCDM-Based Ranking for the diagnosis of HD. A standardized benchmark HD dataset ($I_{HD})\;$from Kaggle (38) was used to evaluate the efficiency of the proposed work. The dataset comprises features extracted from multiple sources, including data from Switzerland $\;(S_{d})$, Hungary $\;(H_{y})$, Cleveland $\;(C_{v})$, and V Long Beach $\;(L_{b}$) as shown in Equation (1).

(1) $$I_{HD}=\;\cup (C_{v},\;H_{y},\;S_{d},\;L_{b})$$

RST analyses the large features set $(I_{HD})\;$ using Johnson's algorithm to generate the minimal subset of attributes $\left(O_{RHD}\right)$ to enhance the classification ability which is shown in Equation (2)

(2) $$O_{RHD}=\;f_{RST}(I_{HD})$$

The selected features $(O_{HD})\;$ are pre-processed with Standardscaler, resulting in the scaled features as shown in Equation (3). This process ensures consistent feature scaling and mitigates the impact of outliers.

(3) $$O_{Rpp}=\;f_{\;pp}(O_{HD})$$

The optimal pre-processed features ($O_{Rpp}$) are classified using various ML classifiers $O_{CL}\;$ including XG, AdaBoost (AB), Logistic Regression (LR), K- Nearest Neighbor (KNN), SVM, Naïve Bayes (NB), Decision Tree (DT), RF, and Extra Trees (ET). This classification process is described in Equation (4).

(4) $$O_{CL}=\;f_{CL}(LR,\;KNN,\;SVM,\;NB,\;DT,\;RF,\;XG,AB,ET)$$

The classification results are evaluated using metrics such as Sensitivity (Sn), F1-score (F1), Specificity (Sp), MCC, MAE, accuracy (Ac), precision (Pr), and training time (Tt). These metrics are represented by $O_{MHD}$ and are given in Equation (5).

(5) $$O_{HD}=\;O_{Rpp}\;(O_{CL\;}(Metrics_{Ac,Pr,Sp,Sn,F1,MCC,\;MAE,Tt\;})$$

To improve the overall performance and robustness of the predictive model, different stacks $\left(O_{s_{i}}\right)$ based on correlation analysis ($O_{CA}$) has been proposed as shown in Equation (6). These stacks $O_{s_{i}}\;$ includes multiple models, resulting in improved accuracy, reduced overfitting, increased flexibility, enhanced stability, and reduced model bias. These stacks $O_{s_{i}}\;$ will be used to evaluate the classification performance of CD.

(6) $$\;\;\;\;\;\;\;\;\;\;\;\;\;\;\;\;\;\;\;\;\;\;O_{s_{i}}=\;f_{s_{i}}(O_{MHD})\;\mathrm{where}\;\;i\;\;\in 1\;\;to\;\;5$$

MCDM offers a robust framework for addressing complex decision-making scenarios involving multiple classification accuracies obtained by the different stacked models $O_{s_{i}}$, often characterized by conflicting criteria. By integrating diverse criteria into the decision-making process, MCDM ensures that decisions are balanced, transparent, and justifiable. In this context, TOPSIS is employed to evaluate and rank the stacks $O_{s_{i}}$, facilitating the identification of the optimal stack ($O_{BS}$) for the accurate prediction of HD, as shown in Equation (7).

(7) $$O_{BS}=f_{MCDM}(O_{s_{i}})$$

**Figure 1:**
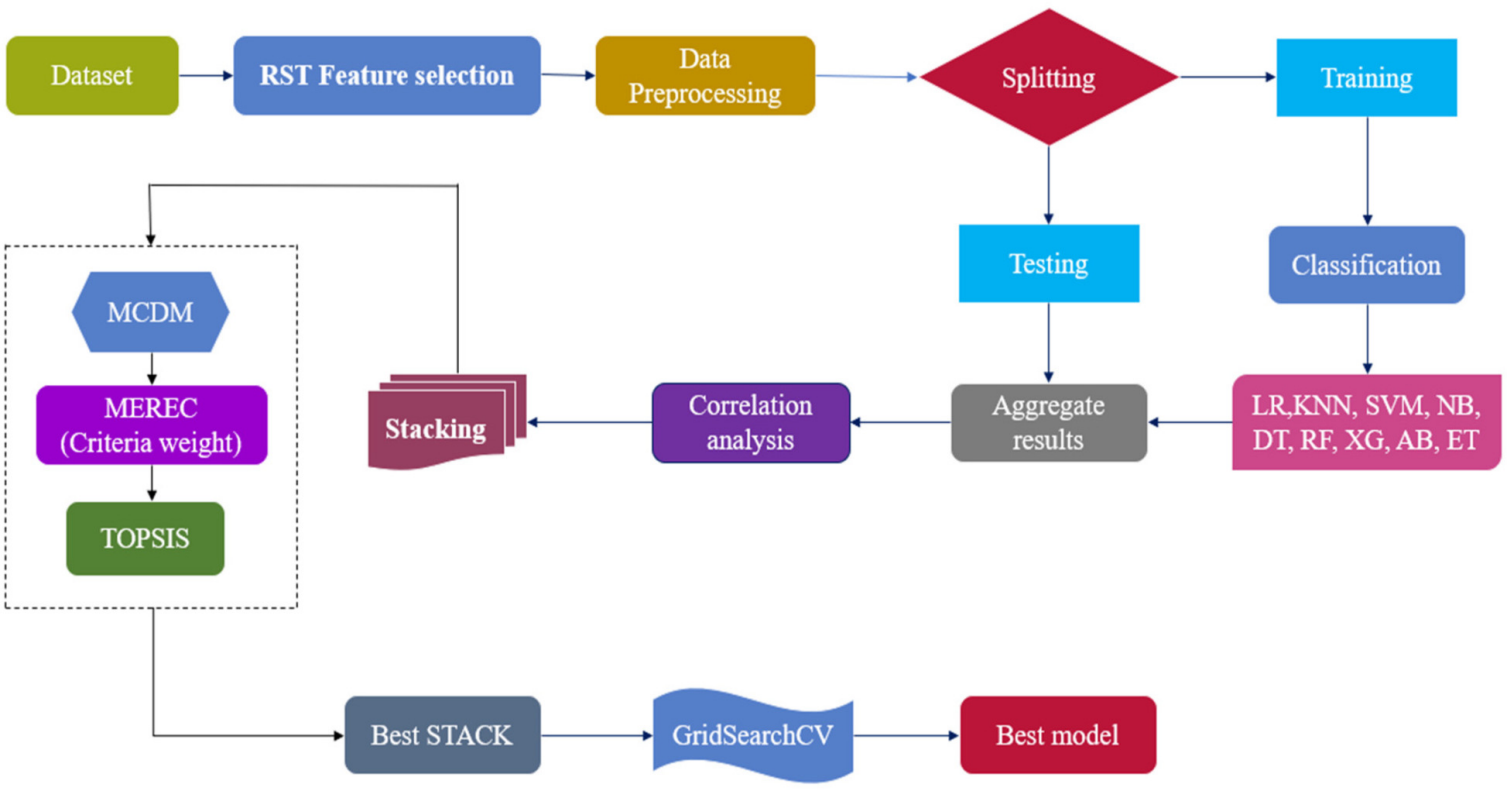
Overall architecture of the proposed work.

The classifiers in the optimal stack $O_{BS}$, are refined using GridSearchCV to optimize hyperparameters by exploring various parameter combinations, ultimately identifying the best classifier $\left(O_{BC}\right)$ for predicting HD. The corresponding equation is provided in Equation (8). The architecture of the proposed optimized RST-ML approch is illustrated in Figure 1.

(8) $$\;O_{BC}=\;f_{Op}(O_{BS})$$

### 2.2 Dataset description

The Kaggle HD dataset ($I_{HD}$), sourced from https://www.kaggle.com/datasets/johnsmith88/heart-disease-dataset, was used in this investigation (38). This dataset aggregates records from four medical institutions: $S_{d}$, $H_{y}$, $C_{v}$, and $L_{b}$. Of the original 76 attributes, a standardized subset of 14 clinically relevant features and a final subset of 303 complete records was selected, primarily from the Cleveland database, which contains no missing values, as adopted in most published studies, to ensure comparability, yielding. This refined dataset improves interpretability and robustness for model training and evaluation. The output label classifies patients into two categories: 1 indicates the presence of cardiac disease, and 0 indicates its absence. These diagnostic labels were determined through clinical evaluation methods, including electrocardiographic results and stress testing. The selected features encompass critical clinical indicators such as age, blood pressure, cholesterol levels, chest pain type, and other parameters commonly used in cardiac risk prediction. A detailed description of these attributes in the HD dataset is provided in Table 1.

**Table 1:** Detailed attribute description of the heart disease dataset.

Feature	Description	Range	Type
Age	Patient's age in completed years.	29–77	Numeric
Sex	Patient's gender (male = 1, female = 0).	0, 1	Nominal
Cp	Type of chest pain: 1 = Typical angina, 2 = Atypical angina, 3 = Non-anginal pain, 4 = Asymptomatic.	1–4	Nominal
Trestbps	Resting blood pressure in mmHg at the time of admission to the hospital.	94–200	Numeric
Chol	Serum cholesterol in mg/dl.	126–564	Numeric
Fbs	Fasting blood sugar > 120 mg/dl (1 = Yes, 0 = No).	0, 1	Nominal
Restecg	Resting electrocardiogram results: 0 = Normal, 1 = ST-T wave abnormality (e.g., T-wave inversion or ST elevation/depression > 0.05 mV), 2 = Left ventricular hypertrophy by Estes’ criteria.	0–2	Nominal
Thalach	Maximum heart rate achieved.	71–202	Numeric
Exang	Exercise-induced angina (0 = No, 1 = Yes)	0, 1	Nominal
Oldpeak	ST depression induced by exercise relative to rest.	0–6.2	Numeric
Slope	Slope of the peak exercise ST segment: 1 = Upsloping, 2 = Flat, 3 = Downsloping.	1–3	Nominal
Ca	Number of major vessels (0–3) colored by fluoroscopy.	0–4	Numeric
Thal	Status of the heart: 3 = Normal, 6 = Fixed defect, 7 = Reversible defect.	3, 6, 7	Nominal
Target	HD diagnosis: 0 = No disease, 1 = Presence of disease	0–1	Nominal

### 2.3 Johnson's algorithm

Numerous applications of RST have shown it to be effective, including data reduction, identifying hidden trends in data, assessing the significance of data, and creating sets of decision rules based on data (39). To identify the most significant features for analysis, Johnson's Algorithm of RST is used. The Johnson's Algorithm also known as Johnson Reducer, uses a simplified greedy method to find a single reduct R. Johnson (40) pointed out that this approach typically finds a minimal-length prime implicant. Until an ideal subset, or reduct, is found, the algorithm iteratively chooses the most important feature that enhances object distinguishability and updates the attribute set. The group of sets associated with the discernibility function is represented by Q, and the weight assigned to each set *Q* in Q is provided by w(Q), which is automatically calculated from the data.

RST was chosen over modern feature selection methods for its ability to handle uncertainty and imprecision without prior assumptions or parameter tuning. It identifies minimal subsets of attributes based on data dependencies, offering high interpretability and transparency which is critical in healthcare applications. Compared to statistical or black-box techniques like Lasso or embedded models, RST provides a rule-based, explainable framework while preserving classification accuracy during dimensionality reduction. This makes RST, and Johnson's Algorithm in particular, a robust and suitable choice for the proposed HD prediction model. Algorithm 1 outlines the steps involved in Johnson's reducer of RST.

**Algorithm 1:** Johnson's Algorithm for finding reducts RST.

**Input**: Information table or Decision Table, I=(U,A)
**Output**: $RED_{A\;}\subseteq A$ of all reducts of A
**Step 1**: Initialize the reduct set *R* as an empty set
R=∅
**Step 2**: Find the attribute *a* that maximizes the sum of weights ∑w(Q), where the sum is taken over all sets *Q* in Q that contain *a*. $$a=\arg max_{a\in Attributes}\underset{\begin{array}{c} Q\in Q\\ a\in Q \end{array}}{\sum }w(Q)$$
**Step 3**: Add attribute *a* to *R* to maximize the weight sum R=R∪{a}
**Step 4**: Remove all sets *Q* from Q that contain *a* to avoid redundant calculation. Q=Q−{Q|a∈Q}
**Step 5**: If Q=∅, return R. If not, return to step 2.

### 2.4 Dataset preprocessing

The significant features $O_{RHD}$, obtained from Johnson's Algorithm of RST was preprocessed using the StandardScaler technique to ensure uniformity in feature scales and improve model training. StandardScaler standardizes data by subtracting the mean and dividing by the standard deviation, as shown in

(9) $$z=\left(\frac{x-\bar{x}}{s}\right)$$

where *x* is the original feature value, $\bar{x}$ is the mean, and *s* is the standard deviation.

This preprocessing step ensures that all features contribute equally to the model, eliminating biases caused by differences in scale. By enhancing convergence during training and mitigating the influence of outliers, StandardScaler improves model performance, resulting in more robust and reliable predictions. The dataset was divided into an 80:20 train-test split ratio, with 80% of the data allocated for training the model and 20% reserved for testing its performance to evaluate and identify the most effective model.

### 2.5 Machine learning models

This section highlights various ML algorithms, each chosen for its unique ability to process diverse data types. ML models have become essential tool for predicting HD due to their capability to analyse complex datasets and uncover valuable insights.

LR offers simplicity and interpretability for binary classification (41) but assumes linear relationships, which can limit its performance on complex data. KNN, a non-parametric method based on proximity (42), is intuitive but sensitive to noise and suffers in high-dimensional spaces. SVM provides robust classification in high-dimensional settings (43), yet is computationally intensive and requires careful kernel tuning. NB, though efficient (44), relies on the unrealistic assumption of feature independence. DT, valued for their transparency (45), are prone to overfitting; hence, ensemble approaches were used to improve generalization. These limitations were addressed using feature scaling, selection, and cross-validation.

To enhance performance and mitigate overfitting, ensemble models including RF, AB, XG, and ET were incorporated. RF reduces variance through bagging (46) but at the cost of interpretability. XG, known for its high accuracy, incorporates L1/L2 regularization (47) but requires extensive tuning. AB adapts to misclassified instances for improved robustness (48), though it is sensitive to noisy labels. ET increases diversity via random splits (49), but excessive randomness may cause instability in small datasets. Each model contributed unique strengths across varying data complexities, allowing a comprehensive evaluation of HD risk prediction (50, 51).

### 2.6 Classifier selection and stacking strategy

The classifiers for stacking were chosen based on Pearson correlation analysis to ensure diversity and complementarity. Five distinct stacks were created, each with specific combinations of classifiers designed to optimize performance. Stack-1 (Hybrid Stack) combines LR, KNN, and SVM, which are highly correlated and offer similar performance. Stack-2 (Tree and Probabilistic Stack) integrates NB, DT, and RF, with NB adding diversity due to its lower correlation with tree models. Stack-3 (Boosted Stack) includes XG, AB, and ET, which are highly correlated to strengthen combined effect, with AB providing unique performance benefits. Stack-4 (Advanced Tree-Based Stack) leverages the high correlations among DT, RF, XG, AB, and ET, maintaining robustness. Stack-5 (Unified Stack) incorporates all nine models to cover a wide range of correlations and performance traits. This stacking approach enhances performance by combining multiple classifiers, allowing the meta-model to improve predictions by learning which base models are most reliable in specific scenarios. Correlation analysis ensures the base model's errors are sufficiently diverse, maximizing the benefits of stacking. The strategy significantly improves prediction accuracy and generalization by leveraging the complementary strengths of various classifiers, thus enhancing the overall model's robustness.

### 2.6.1 Correlation analysis

Correlation analysis, denoted as $O_{CA}$, measures the linear relationship between predictions from different classifiers to ensure diversity in stacking. The proposed work utilizes Pearson correlation to identify and group classifiers based on their performance similarity, aiming to balance diversity and complementarity in each $O_{S_{i}}$ for optimal performance. The procedure for correlation analysis is detailed in Algorithm 2.

**Algorithm 2:** Grouping ML models as Stacks based on $O_{CA}$.

**Input**: RST – ML metrics ($O_{MHD}$)
**Output**: Different stacks ($O_{S_{i}})$
**Step 1**: Imputation of Performance metrics as matrix
$X=M_{ij},$ where 1≤i≤9&1≤j≤8
where *i* represents model and *j* represents performance metrics.
**Step 2**: Compute Correlation Coefficients
***Step 2.1***: Covariance Calculation $Cov(X_{i},\;X_{j})=\;\frac{\sum_{k=1}^{n}(X_{ik}-\;\bar{X_{i}})(X_{\;jk}-\;\bar{X_{j}})}{n-1}$
***Step 2.2***: Variance Calculation $Var\;(X_{i})=\;\frac{\sum_{k=1}^{n}{\left(X_{ik}-\;\bar{X_{i}}\right)}^{2}}{n-1}$ $Var\;(X_{j})=\;\frac{\sum_{k=1}^{n}{\left(X_{\;jk}-\;\bar{X_{j}}\right)}^{2}}{n-1}$
***Step 2.3***: Pearson Correlation Coefficient $r_{ij}=\;\frac{Cov(X_{i},\;X_{j})}{\sqrt{Var\;(X_{i})\text{·}\;Var\;(X_{j})}}$
**Step 3**: Compute Correlation Matrix $CM=\;C_{ij},\;where\;1\leq i\leq 8\;\&\;1\leq j\leq 8$
***Step 3.1***: Interpretation
*Step 3.1 (a):* High Positive Correlation (0<c≤1)
*Step 3.1 (b):* Zero Correlation (c=0)
*Step 3.1 (c)*: Negative Correlation (−1≤r<0)
**Step 4**: Group Classifiers into stacks based on Correlation
$O_{S_{i}}$, where 1≤i≤5

#### 2.6.2 Stacked classifier

Stacking, or stacked classifiers (52), is an ensemble learning technique that combines several Base Classifiers (BC) through a Meta-Classifier (MC) to improve performance. This approach trains multiple models on the same data and uses their predictions as inputs for a meta-model, which leverages the strengths of each model while mitigating its weaknesses. The principle is that no single algorithm is optimal for all problems. If $\left\{h_{1},\;h_{2},\;\ldots ,\;h_{n}\right\}$ are base classifiers and *H* is the meta-classifier, the final prediction $\hat{y}\;$ is given by Equation (10)

(10) $$H\;(h_{1}(x),\;h_{2}(x),\;\ldots ,\;h_{n}\;(x))$$

where *x* is the input feature vector.

**Figure 2:**
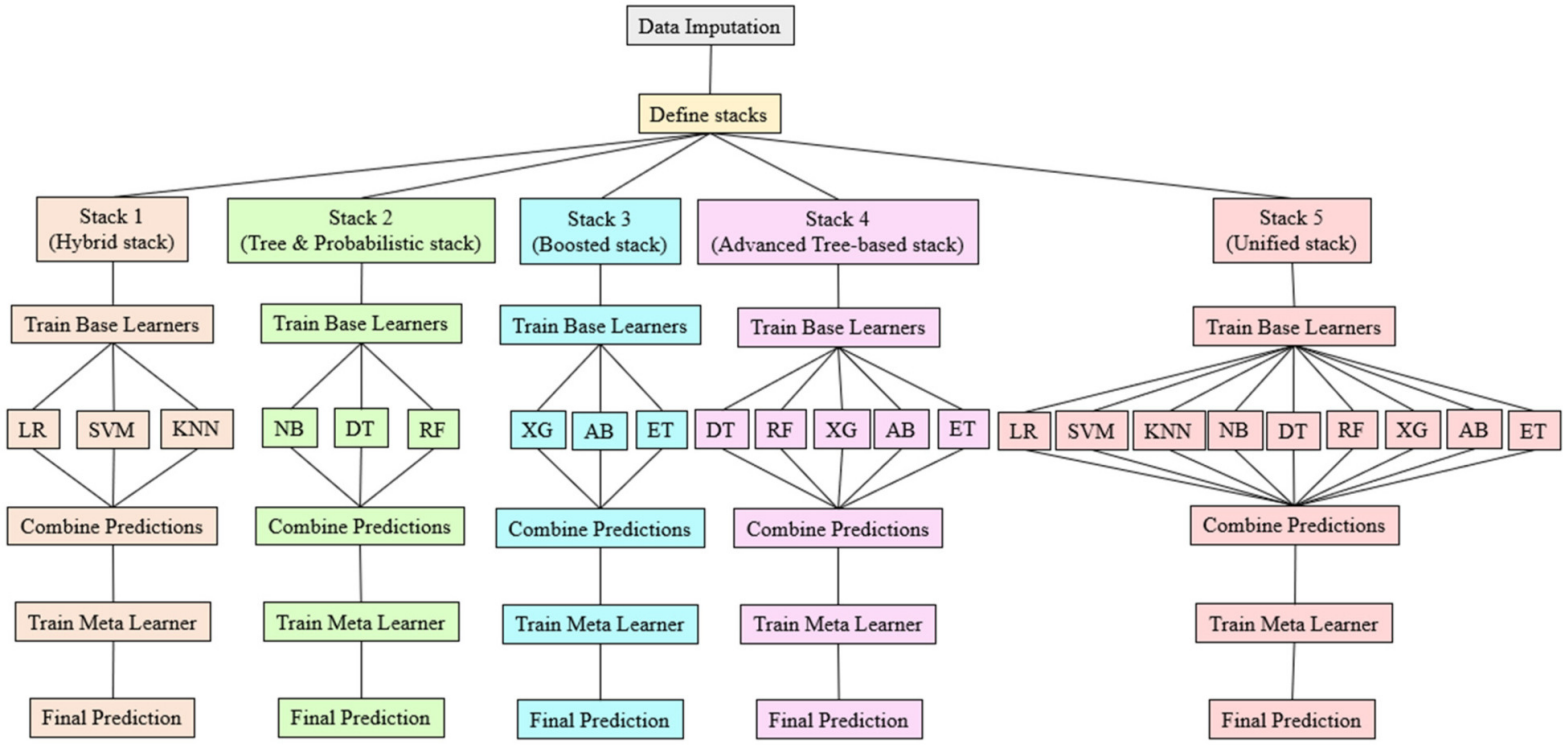
Overview of stacking classifier.

In the proposed work, different stacks $O_{S_{i}}$ is employed to identify the best prediction model for predicting HD. Figure 2 illustrates each phase of the $O_{S_{i}}$, detailing the process from base model training to final predictions.

The Algorithm 3 provided below outlines the sequential steps involved in combining the predictions of multiple base models to produce the final output.

**Algorithm 3:** Performance evaluation of Stacking classifiers.

Input: RST preprocessed dataset ($O_{Rpp}$)
Output: Performance of Stacked classifiers ($O_{S_{i_{MHD}}}$)
**Step 1**: Data Imputation $X=\;O_{Rpp}$
**Step 2**: Split Dataset $X_{S}=(X_{train},\;X_{test},\;y_{train},\;y_{test})=S(X,\;y,\;0.2,\;RS=42)$
**Step 3**: Define Stacks
***Step 3.1***. Stack 1 – Hybrid Stack $BC_{1}=\{LR_{O_{Rpp}},\;KNN_{O_{Rpp}},\;SVM_{O_{Rpp}}\};\;MC_{1}=\;M_{meta,1}$
***Step 3.2.*** Stack 2 – Boosted Stack $BC_{2}=\{\;NB_{O_{Rpp}},\;DT_{O_{Rpp}},\;RF_{O_{Rpp}}\};\;MC_{2}=\;M_{meta,2}$
***Step 3.3***. Stack 3 – Tree and Probabilistic stack $BC_{3}=\{XG_{O_{Rpp}},\;AB_{O_{Rpp}},\;ET_{O_{Rpp}}\};\;MC_{3}=\;M_{meta,3}$
***Step 3.4***. Stack 4 – Advanced tree-based stack $BC_{4}=\{\;DT_{O_{Rpp}},\;RF_{O_{Rpp}},\;XG_{O_{Rpp}},\;AB_{O_{Rpp}},\;ET_{O_{Rpp}}\};\;MC_{4}=\;M_{meta,4}$
***Step 3.5***. Stack 5 – Unified stack $BC_{5}=\{LR_{O_{Rpp}},KNN_{O_{Rpp}},SVM_{O_{Rpp}},NB_{O_{Rpp}},DT_{O_{Rpp}},RF_{O_{Rpp}},XG_{O_{Rpp}},AB_{O_{Rpp}},ET_{O_{Rpp}}\};$
$$MC_{5}=\;M_{meta,5}$$
**Step 4**: Train and evaluate each stack $S_{i}$ (i=1to5)
***Step 4.1***: BC $${\hat{Y}}_{train,i}=[CL_{i,j}(X_{train})\;for\;j\;in\;BC_{i}];\;{\hat{Y}}_{test,i}=[CL_{i,j}(X_{test})\;for\;j\;in\;BC_{i}]$$
***Step 4.2***: Combine predictions from BC $X_{meta,train,i}=\;{\hat{Y}}_{train,i};\;X_{meta,test,i}=\;{\hat{Y}}_{test,i}$
***Step 4.3***: Train MC $M_{meta,i}\;.\;fit\;(X_{meta,train,i},\;y_{train})$
**Step 4.4**: Predict using MC ${\hat{Y}}_{meta,i}=M_{meta,i}\;.\;predict\;(X_{meta,train,i})$
**Step 5**: Performance evaluation ${S_{i}}_{MHD}=f_{Ymeta,i}(Metrics{\;}_{Acc,Pr,Rc,F1,MCC,\;MAE,Tt})$

### 2.7 MCDM ranking

To refine the selection of the best-performing stack $O_{BS}$, a MCDM approach is implemented, addressing the challenge of repeated accuracy values across stacked classifiers $O_{S_{i}}$. By incorporating multiple evaluation metrics including Sn, F1, Sp, MCC, MAE, Ac, Pr and Tt, a comprehensive and balanced assessment is ensured. MCDM enables a detailed comparison across these criteria, fostering robust and well-informed decision-making.

Although AUC is a commonly used and intuitive metric in healthcare for measuring a model's ability to discriminate between classes, it captures only one dimension of performance. The proposed work employs MCDM ranking to evaluate models across multiple metrics simultaneously, including Acc,Pr,Rc,F1,MCC,MAEandTt. This approach provides a comprehensive evaluation framework that accounts for various aspects of model performance, reflecting the complex trade-offs often encountered in clinical decision-making. For example, a model with a high AUC might have suboptimal recall or precision, which could have important implications in patient diagnosis or treatment.

To facilitate understanding among healthcare professionals, the MCDM rankings was presented alongside detailed explanations of each contributing metric, thereby ensuring transparent and actionable insights that extend beyond the scope of AUC alone. Specifically, the TOPSIS method is used for ranking the stacks, and the MEREC method is employed for objective criteria weighting.

#### 2.7.1 MEREC weighting method

The MEREC method, introduced by Keshavarz-Ghorabaee et al., calculates criteria weights by evaluating the influence of each criterion on the performance of alternatives (53). This method assigns higher weights to criteria with a more significant impact, ensuring an objective weighting process. The steps of MEREC are outlined in Algorithm 4:

**Algorithm 4:** MCDM Criteria weight calculation.

**Input**: $S_{i_{MHD}}$as Decision matrix (dm)
**Output**: Criteria weight ($w_{r}$)
**Step 1**: Establish the Principal dm $$dm=[s_{tr}]=\;\left[\begin{array}{ccc} s_{11} & \ldots & s_{1r}\\ \ldots & \ldots & \ldots \\ s_{t1} & \ldots & s_{tr} \end{array}\right]\;,\;where\;\;1\leq t\leq m\;,\;$$
1≤r≤n
**Step 2**: Normalization of dm $N_{tr}=\frac{\min x_{tr}}{x_{tr}},\;if\;r\;is\;a\;beniteficial\;creria$ $N_{tr}=\frac{x_{tr}}{\max x_{tr}},\;if\;r\;is\;a\;non-beniteficial\;creria$
**Step 3**: Determine the Alternative's Overall Performance ($P_{t}$) $$P_{t}=\ln\left(1+\;\left(\frac{1}{m}\;\underset{r}{\sum }|\ln(n_{tr})|\right)\right)$$
**Step 4**: Re-evaluate the performance of each $P_{t}$ by excluding the impact of each criterion. $$P_{tr}^{\prime }=\;\ln\left(1+\;\left(\frac{1}{m}\;\underset{l,l\neq r}{\sum }|\ln(n_{tr})|\right)\right)\;$$
**Step 5**: Calculate Absolute Deviations (Ad) $Ad_{r}=\underset{t}{\sum }|P_{tr}^{\prime }-\;P_{t}\;|$
**Step 6**: Determine Criteria Weights ($w_{r}$)
$w_{r}=\frac{Ad_{r}}{\sum_{r}Ad_{r}}$

#### 2.7.2 TOPSIS alternative assessment

Once the criteria weights ($w_{r}$) are determined using MEREC, the stacks $O_{S_{i}}$ are ranked using the TOPSIS method. Developed by Hwang and Yoon in 1981, TOPSIS evaluates alternatives based on their proximity to an ideal solution (positive ideal) and their distance from a worst-case scenario (negative ideal) (54). This ensures that the chosen Stack $O_{BS}$ exhibits optimal performance across all criteria. The steps of TOPSIS, are outlined in Algorithm 5:

**Algorithm 5:** Ranking Alternatives based on TOPSIS.

**Input**: $S_{i_{MHD}}$ as Decision matrix (dm)
**Output**: Rank r
**Step 1**: Establish dm $$dm=[s_{tr}]=\;\left[\begin{array}{ccc} s_{11} & \ldots & s_{1r}\\ \ldots & \ldots & \ldots \\ s_{t1} & \ldots & s_{tr} \end{array}\right]\;,\;where\;\;1\leq t\leq m\;,\;$$
1≤r≤n
Step 2: Normalizing dm (Ndm) with Vector Normalization $r_{tr}=\frac{x_{tr}}{\sqrt{\sum_{t=1}^{m}x_{tr}^{2}}}$
Step 2: Compute the weighted Ndm. $u_{tr}=\;r_{tr}\times w_{r}\;\;,\;where\;\;1\leq t\leq m\;,\;1\leq r\leq n$ $$U=\;\left[\begin{array}{ccc} u_{11} & \ldots & u_{1n}\\ \ldots & \ldots & \ldots \\ u_{m1} & \ldots & u_{mn} \end{array}\right]$$
Step 3: Identify the positive ($A^{+}$) and negative ($A^{-}$) ideal solutions. $$A^{+}=\{(\max u_{tr}|\;r\in R),\min v_{tr}|\;r\in \;R^{\prime }\}$$ $$A^{-}=\{(\min u_{tr}|\;r\in R),\max v_{tr}|\;r\in \;R^{\prime }\}$$
Step 4: Calculate separation measures of positive ($S_{t}^{+}$) and negative ($S_{t}^{-}$) ideal solutions. $$S_{t}^{+}=\sqrt{\overset{m}{\underset{t=1}{\sum }}{\left(u_{tr}-u_{r}^{+}\right)}^{2}}$$ $$S_{t}^{-}=\sqrt{\overset{m}{\underset{t=1}{\sum }}{\left(u_{tr}-u_{r}^{-}\right)}^{2}}$$
Step 5: Determine the relative closeness from the ideal Solution ($C_{t}$) $$C_{t}=\frac{S_{t}^{-}}{\left(S_{t}^{-}+\;S_{t}^{+}\right)}$$
Step 6: Rank (r) the alternatives according to preference order.

### 2.8 Gridsearchcv for hyperparameter tuning

**Figure 3:**
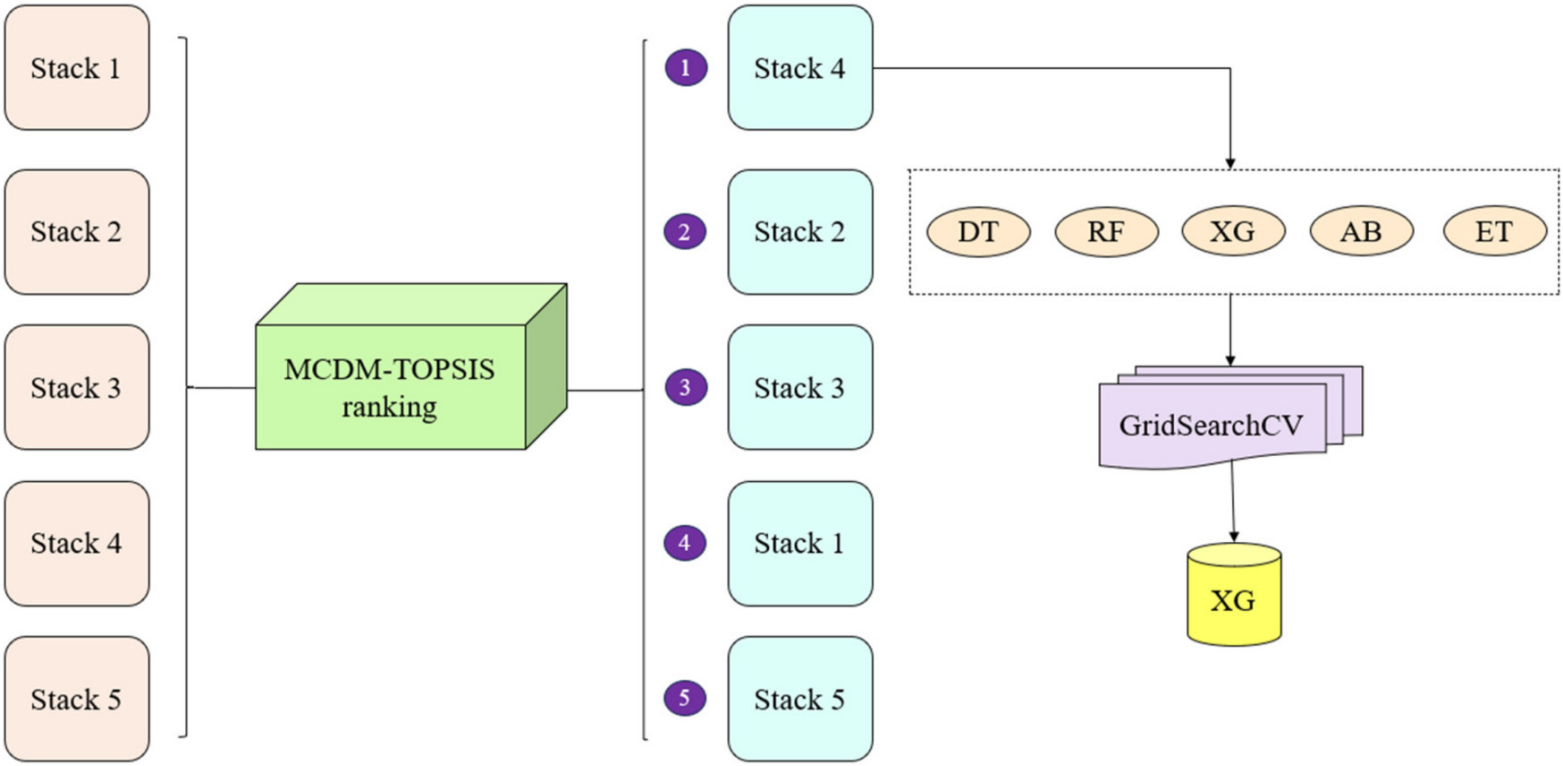
Ranking and optimization of stacked classifiers using MCDM-TOPSIS and gridSearchCV.

To identify best classifier $O_{BC}$ for HD prediction, the top-performing stack $O_{BS}$, determined through MCDM-TOPSIS ranking, undergoes hyperparameter optimization using GridSearchCV (55). GridSearchCV systematically explores predefined hyperparameter ranges for each classifier within $O_{BS}$, optimizing parameters such as learning rate, tree depth, and regularization strength. Leveraging CV, it evaluates parameter combinations to balance overfitting and underfitting, ensuring robust model performance. Each classifier in $O_{BS}$ is fine-tuned individually, and the one achieving the highest validation score post-tuning is selected as $O_{BC}$. This comprehensive process ensures that the best-performing classifier is identified, optimized, and validated, resulting in a reliable and accurate predictive model for HD prediction. The detailed process of ranking and optimization of stacked classifiers using MCDM-TOPSIS and GridSearchCV is provided in Figure 3.

### 2.9 Statistical Analysis

The model's performance is assessed using various standard metrics, including Ac,Pr,Sn,Sp,F1,MCC,MAE and Tt. To compare the performance across different classifiers, statistical tests such as paired *t*-tests and Wilcoxon signed-rank tests are applied.

## 3 Research findings

This section presents the key findings of the study, including RST-based feature selection using Johnson's algorithm, evaluations of ML models $O_{CL\;},$ performance analysis of stacked models $O_{S_{i}}$, MCDM-TOPSIS ranking, and GridSearchCV optimization of the top ranked stack $O_{BS}$.

### 3.1 Experimental setup

The experiments were conducted using ROSETTA for RST feature selection, Python for ML model development, and MATLAB for MCDM-TOPSIS ranking. The model training and optimization were performed in Google Colab, a cloud-based Jupyter notebook environment, utilizing PyTorch for ML implementation. The system used for training was equipped with a 12th Gen Intel Core i3-1215U processor (1.20 GHz) and 8 GB RAM, ensuring efficient data processing and optimal performance for HD prediction.

### 3.2 Correlation heat map of HD dataset

**Figure 4:**
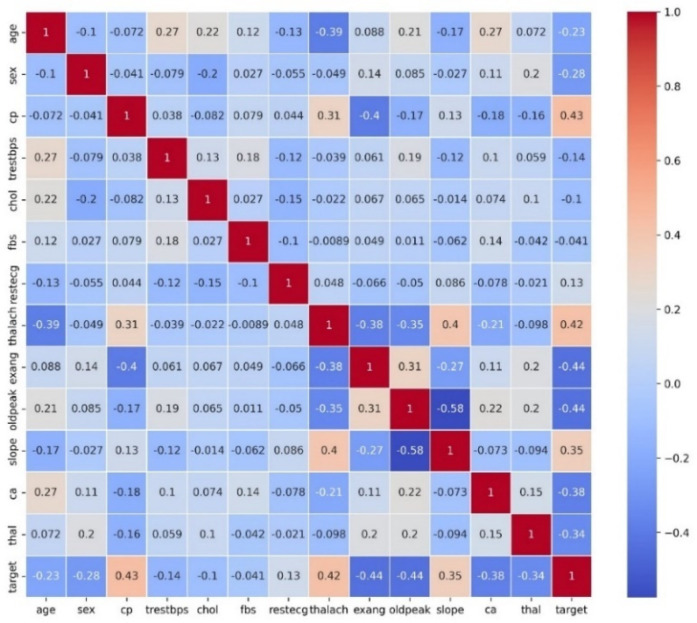
Correlation matrix heatmap of the HD dataset.

Figure 4 displays a heat map which presents a correlation matrix of the features in the HD dataset. The correlations between feature pairs are shown visually in this depiction, where the color intensity corresponds to the direction and strength of the correlation coefficients. This heat map serves as a valuable tool for exploring and understanding the intricate relationships between different features in the dataset, guiding subsequent data preprocessing and modeling steps.

### 3.3 Feature selection using rough set theory

Feature selection in this study was carried out using RST, which utilizes the concept of reducts to identify the most important features. The aim of this process was to reduce the dimensionality of the dataset while preserving the critical attributes needed to predict HD. To achieve this, Johnson's algorithm is applied within the ROSETTA software, a widely recognized method in RST for identifying the minimal set of features that maintain the decision-making capability of the dataset. The original dataset contained 14 features, but after applying RST-based feature selection, only three features namely, Age, resting blood pressure and serum cholesterol was selected. This reduction in features simplifies the model, improves computational efficiency, and lowers the risk of overfitting, while maintaining the essential predictive power. These selected features were then used for model training and evaluation, ensuring that the model focused on the most influential variables for accurate HD prediction.

### 3.4 Model performance evaluation for HD prediction using Ml

After selecting the most significant features through RST, the dataset was preprocessed and used to train several ML models, $O_{CL\;}$. The performance of these models is presented in Table 2 which provide valuable insights for further model enhancement through techniques like hyperparameter tuning and stacking.

**Table 2:** Results of ML models.

S. No.	Model	$A_{c}$	$P_{r}$	$S_{p}$	$S_{n}$	$F_{1}$	MCC	MAE	$T_{t}$
1	LR	0.62	0.65	0.64	0.60	0.63	0.24	0.38	3.28
2	KNN	0.90	0.93	0.92	0.88	0.91	0.81	0.10	0.16
3	SVM	0.61	0.66	0.69	0.54	0.59	0.23	0.39	0.31
4	NB	0.63	0.68	0.69	0.58	0.63	0.27	0.37	0.04
5	DT	0.98	0.98	0.97	0.98	0.98	0.95	0.02	0.37
6	RF	0.98	0.98	0.97	0.98	0.98	0.95	0.02	13.3
7	XG	0.92	0.89	0.87	0.97	0.93	0.85	0.08	2.16
8	AB	0.73	0.74	0.71	0.75	0.75	0.46	0.27	4.56
9	ET	0.98	0.98	0.97	0.98	0.98	0.95	0.02	7.03

### 3.5 Enhancing HD prediction through stacking

**Figure 5:**
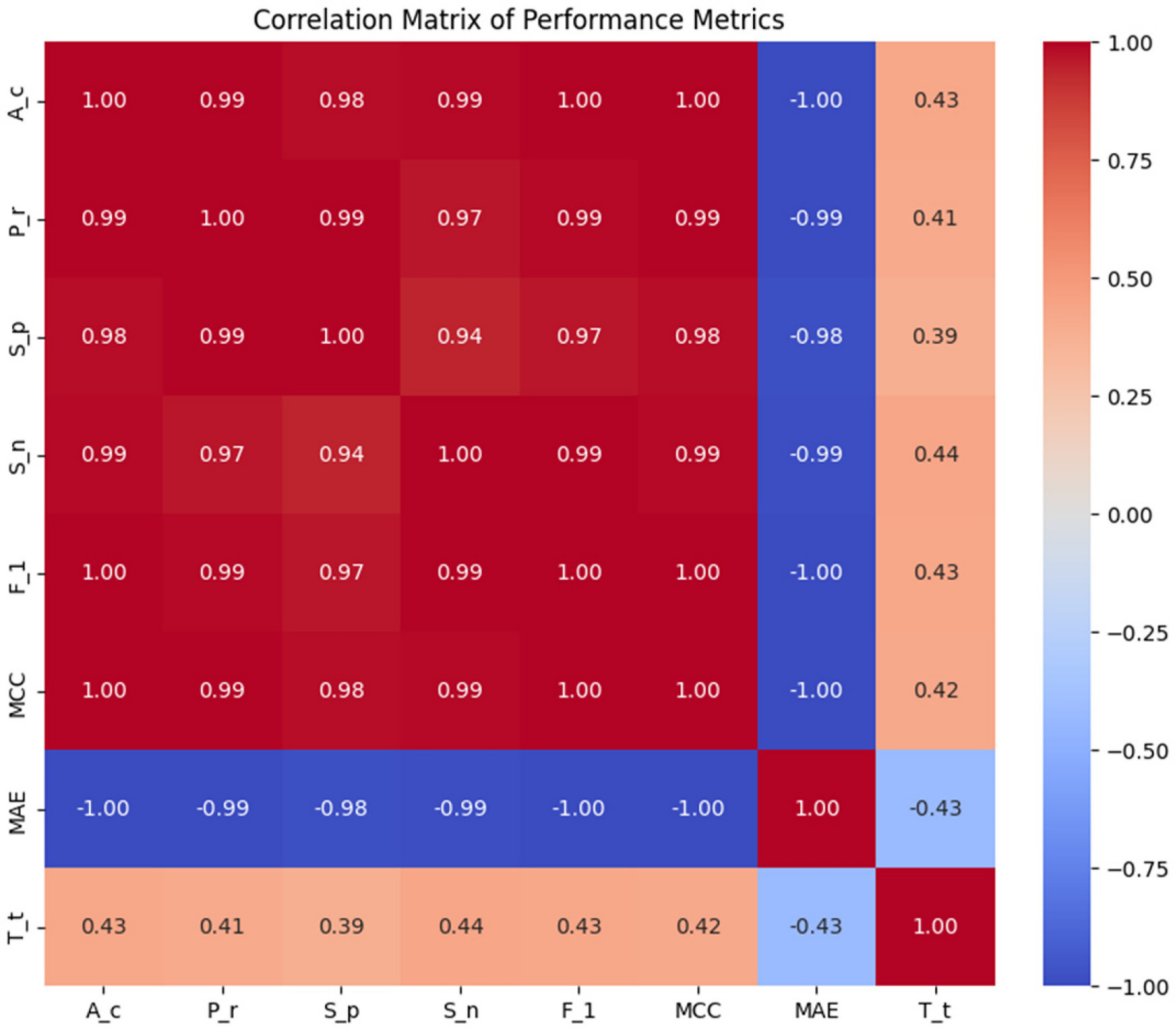
Pearson correlation matrix of the performance metrics.

To improve HD prediction accuracy, stacked ensemble learning was applied. The base learners for these stacking models were carefully selected through Pearson correlation analysis $O_{CA}$, ensuring a balance between diversity and complementarity. Figure 5 presents the Pearson Correlation Matrix, illustrating performance relationships across models, while Table 3 shows the results of stacking classifiers that combine multiple ML models.

**Table 3:** Performance evaluation of the stacking classifiers.

Model	$A_{c}$	$P_{r}$	$S_{p}$	$S_{n}$	$F_{1}$	MCC	MAE	$T_{t}$
Stack – 1	0.75	0.74	0.79	0.70	0.76	0.49	0.25	0.17
Stack – 2	0.98	0.96	1.00	0.96	0.98	0.96	0.02	1.33
Stack – 3	0.98	0.96	1.00	0.96	0.98	0.96	0.02	1.83
Stack – 4	0.98	0.96	1.00	0.96	0.98	0.96	0.02	0.59
Stack – 5	0.98	0.96	1.00	0.96	0.98	0.96	0.02	3.16

### 3.6 MCDM –TOPSIS approach

The results from the stacking models in Table 3 shows that Stack-2 to Stack-5 exhibit very similar performance across most metrics, while Stack-1 has lower performance across all metric. The TOPSIS-based MCDM technique was employed to identify the top-performing stack. Table 3 serves as the primary decision for the analysis. Weights were assigned to each criterion using the MEREC method, which measures the impact of removing a criterion on overall performance. This approach ensures that the weight assignment reflects the significance of each criterion in the decision-making process. The criteria weights determined by the MEREC approach are shown in Table 4.

**Table 4:** Criteria Weights determined by the MEREC Method.

Method	$A_{c}$	$P_{r}$	$S_{p}$	$S_{n}$	$F_{1}$	MCC	MAE	$T_{t}$
MEREC	0.0669	0.0683	0.0732	0.0574	0.0695	0.0496	0.2264	0.3685

**Figure 6:**
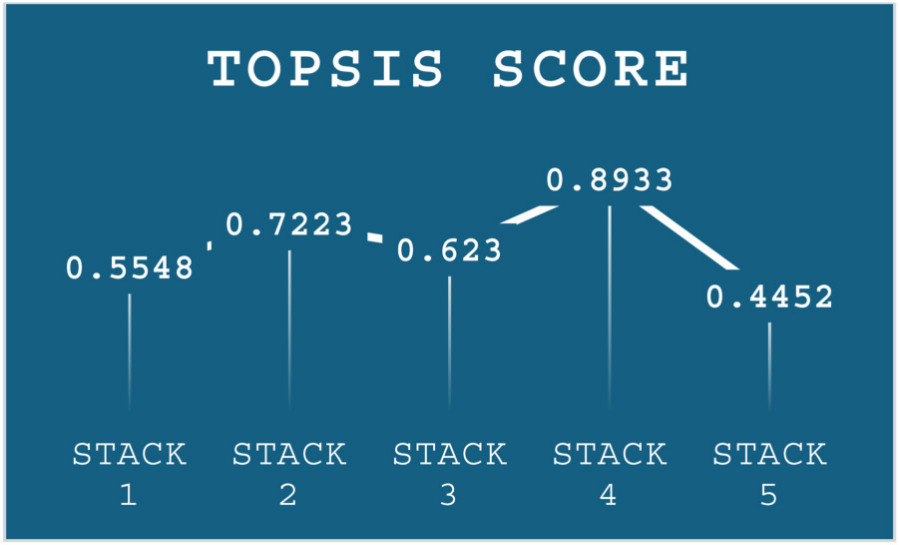
Performance score of TOPSIS.

These weights were then incorporated into the weighted normalization process, forming the foundation for the subsequent TOPSIS steps. The results of the TOPSIS analysis, depicted in Figure 6, reveal that Stack-4 stands out as the optimal model, achieving the highest score of 0.8933. This score reflects its superior predictive performance and robustness compared to the other stacks.

### 3.7 Hyperparameter tuning and performance evaluation of stack 4

To find the best ML classifier, $O_{BC}$, from Stack – 4, GridSearchCV is utilized to tune hyperparameters. This method systematically explores various combinations and uses CV to evaluate performance. By identifying the optimal hyperparameters, GridSearchCV ensures selection of the best-performing ML model. The results of this comprehensive evaluation are presented in Table 5.

**Table 5:** Performance metrics of ML models in STACK- 4 tuned with gridSearchCV.

Model	$A_{c}$	$P_{r}$	$S_{p}$	$S_{n}$	$F_{1}$	MCC	MAE	$T_{t}$
DT	0.9773	0.9756	0.9816	0.9724	0.9786	0.9544	0.0227	0.003
RF	0.9773	0.9756	0.9816	0.9724	0.9786	0.9544	0.0227	0.19
XG	**0**.**9870**	**0**.**9760**	**1**.**00**	**0**.**9724**	**0**.**9879**	**0**.**9742**	**0**.**0129**	**0**.**04**
AB	0.7825	0.7892	0.8037	0.7586	0.7964	0.5630	0.2175	4.56
ET	0.9773	0.9756	0.9816	0.9724	0.9786	0.9544	0.0227	7.03

The bold values highlight the best results, obtained by XG, across each evaluation metric.

**Figure 7:**
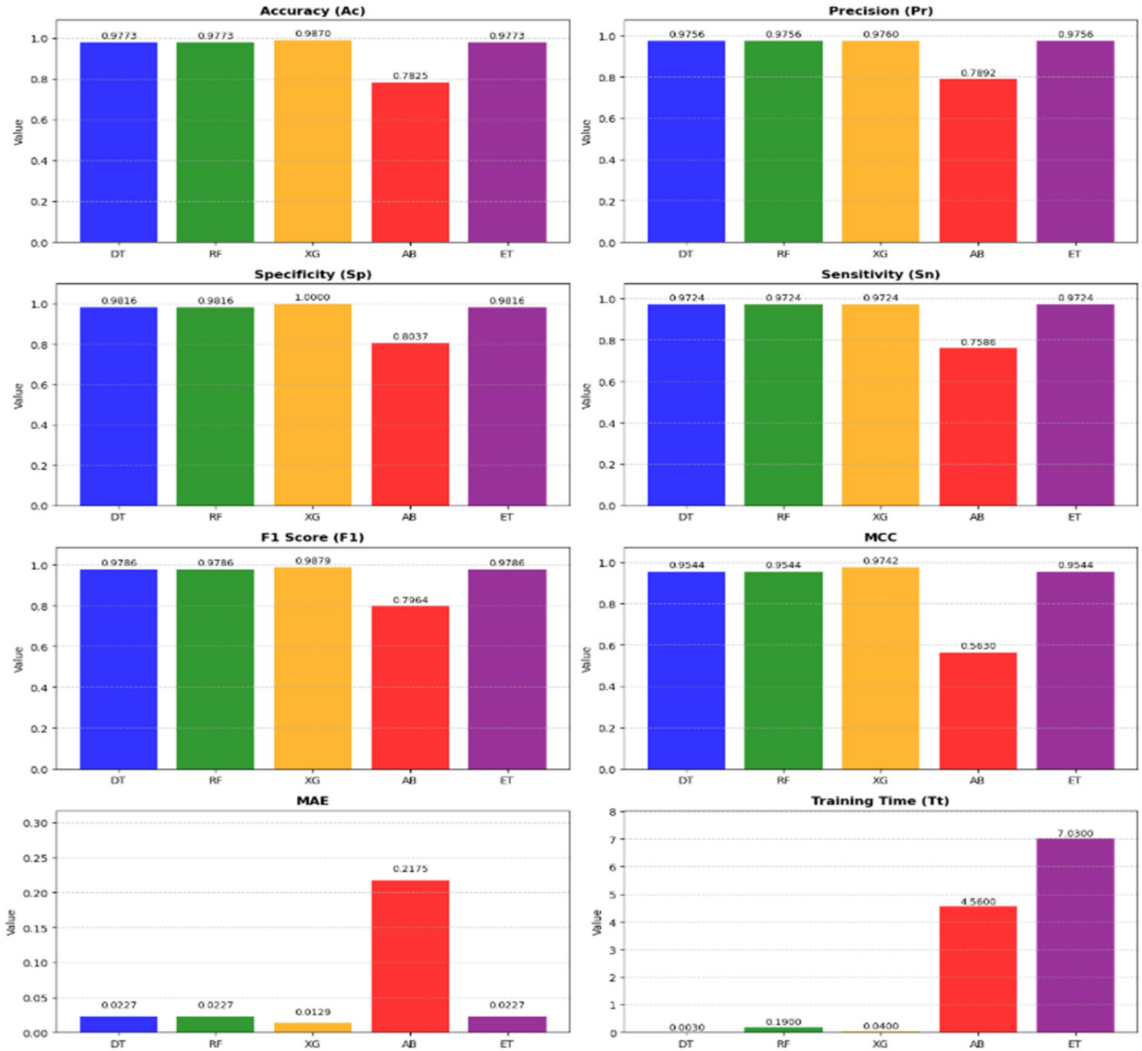
Metrics-wise performance comparison of stack-4 models.

Figure 7 presents a detailed comparison of the metrics across the models in Stack-4, underscoring the effectiveness in identifying the top classifier. It shows that XG is the highest-performing model among other models in Stack 4.

### 3.8 Statistical and practical evaluation of model performance

To assess the statistical significance of performance differences between the models, paired *t*-tests and Wilcoxon Signed-Rank tests was conducted. The results revealed no statistically significant differences, with all *p*-values being greater than 0.05. While trends were observed, such as a *p*-value of 0.09097 for the comparison between DT and XG, these were not strong enough to reject the null hypothesis. From a practical standpoint, XG excelled in terms of $A_{c}$, $F_{1}$, MCC, and MAE. Moreover, XG demonstrated an impressive $T_{t}$ of just 0.04 s, considerably faster than other models. Although the statistical tests showed no significant differences between the models, the superior performance of XG, coupled with its fast $T_{t}$, makes it the ideal choice for this task. Therefore, the final model selection is influenced by performance metrics and the practical considerations such as training efficiency and ease of interpretation, with XG standing out as the optimal model.

### 3.9 Interpretability of the prediction model using SHAP and LIME

After selecting XG as the best model for HD prediction, SHAP and LIME is used for model interpretability to ensure its decisions are based on the right features. SHAP's summary, dependence, and force plots highlight key features and their impact on predictions, while LIME's bar plots provide detailed explanations for individual predictions, especially in edge cases. These techniques help validate the model's behaviour, ensuring it aligns with domain knowledge and can be trusted for real-world applications.

#### 3.9.1 SHAP interpretation

**Figure 8:**
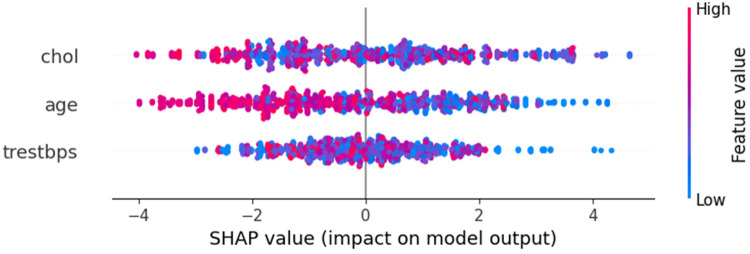
SHAP summary plot.

The SHAP summary plot in Figure 8 provides a concise overview of feature importance across all data points. It ranks features by their influence on the model's predictions for HD risk, with “chol” as the most impactful, followed by “age” and “trestbps”. Each point represents a sample, with the *x*-axis showing the SHAP value, indicating the feature's contribution to risk: positive values increase risk, while negative values decrease it. The color gradient reflects feature values, highlighting trends like higher cholesterol levels being strongly associated with increased risk.

**Figure 9:**
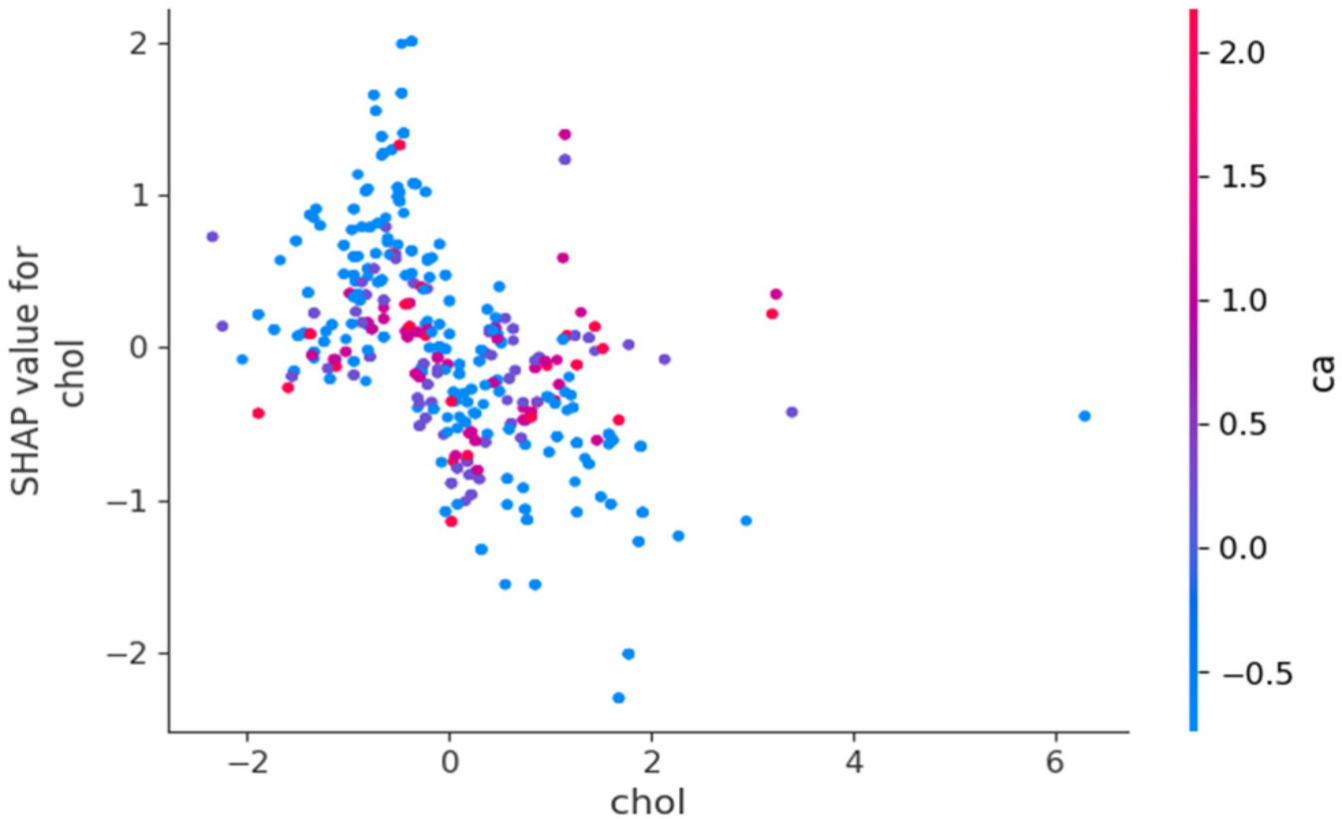
SHAP dependence plot for cholesterol.

Figure 9 reveals a clear negative correlation between cholesterol levels and their impact on model predictions. As cholesterol values increase along the *x*-axis, their corresponding SHAP values generally decrease on the *y*-axis, indicating that higher cholesterol levels contribute more strongly to predicting HD risk. Lower cholesterol values tend to have positive SHAP values, suggesting they decrease HD risk, while higher cholesterol values generally have negative SHAP values, indicating increased risk. The vertical spread of points at each cholesterol level and the variation in colours further emphasizes that the relationship between cholesterol and HD prediction is complex and moderated by other factors in the model.

**Figure 10:**
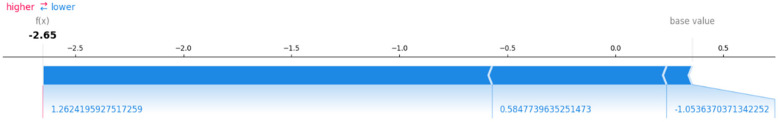
SHAP force plot for a single prediction.

SHAP force plot explains the contribution of each feature to a single prediction. It highlights how each feature contributes positively or negatively to the model's prediction, allowing users to understand why a particular instance is classified as “high risk” or “low risk” for HD. The force plot shows the aggregated contribution of each feature towards the model's final prediction, which is crucial for interpreting individual predictions. Figure 10 explains a single prediction, showing how the model's base value of 0.5 is adjusted to a final output of −2.65 by three feature contributions. The blue segments represent features lowering the HD risk, with their cumulative effect resulting in a low-risk prediction. This plot provides a clear, quantitative breakdown of how each feature impacts the final prediction.

#### 3.9.2 LIME interpretation

**Figure 11:**
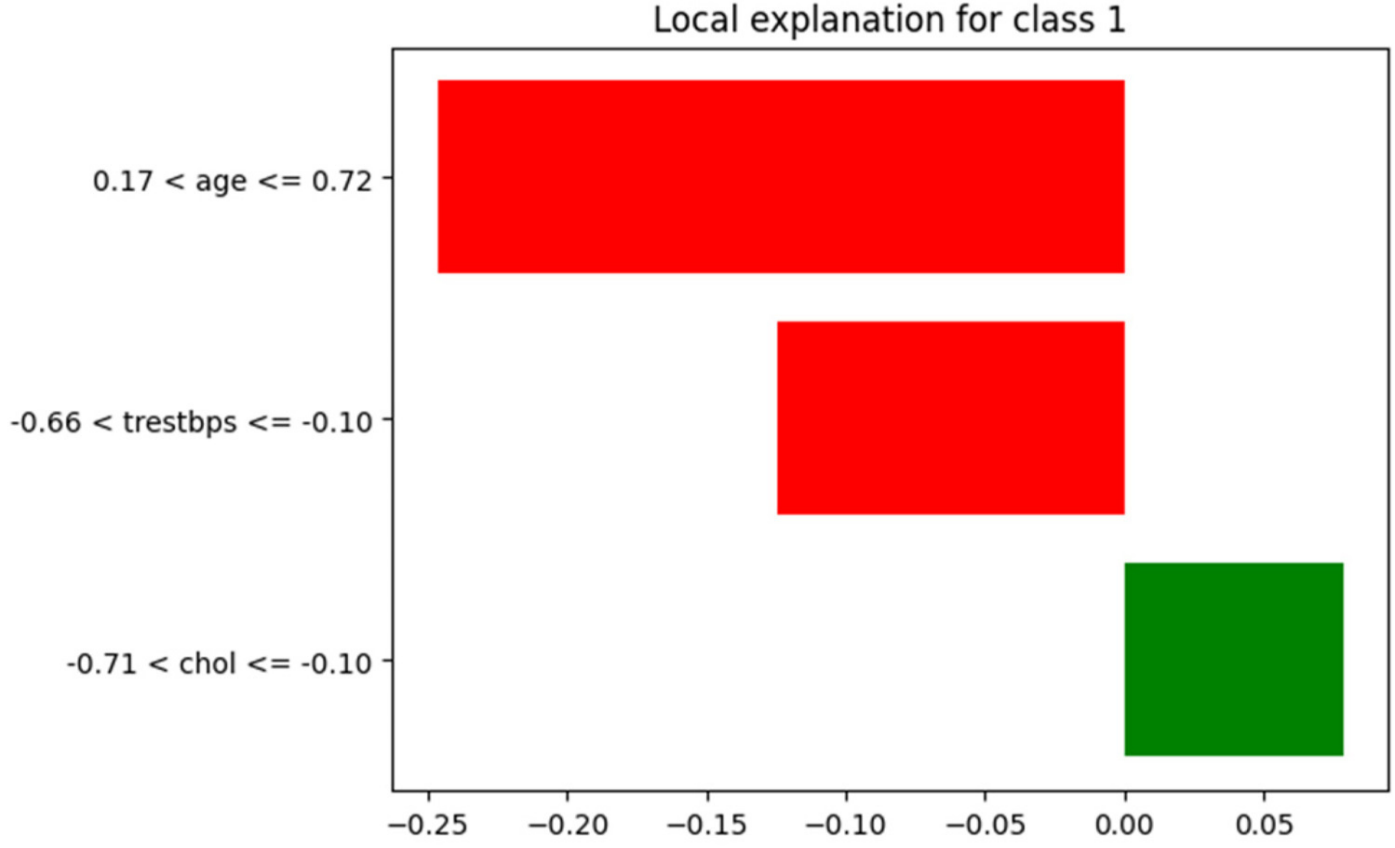
LIME bar plot for individual prediction.

LIME offers a local explanation for a single prediction, showing the importance of features for that specific prediction. In bar plots, features are ranked according to their contribution to the prediction, which can be particularly useful in understanding the reasons behind misclassifications. Figure 11 highlights the contributions of three features to a specific HD prediction. Age has the strongest negative impact (−0.2462), significantly reducing risk, followed by blood pressure with a moderate negative effect (−0.1243). Cholesterol contributes slightly positively (+0.0786), marginally increasing risk. The normalized feature ranges and impact magnitudes (−0.25 to 0.05) collectively point to a low-risk classification, offering a clear, interpretable explanation for this individual prediction.

### 3.10 Performance evaluation of proposed model with state-of-the-art models

A comprehensive comparison of state-of-the-art techniques for HD prediction is presented, highlighting their methodologies, strengths, limitations, and accuracies. These approaches range from traditional ML models to advanced hybrid techniques, with a particular focus on feature selection strategies and dataset utilization. Notably, the proposed Optimized RST-ML model demonstrates superior accuracy, combining RST, ML, stacked ensemble, and MCDM. This hybridized approach, which has not been previously proposed for HD prediction in the literature, offers a novel and robust solution for HD detection and clinical decision-making. The detailed comparison of these techniques is provided in Table 6.

**Table 6:** Comparison of the proposed model with state-of-the-art models for HD prediction.

S. No.	Reference	Technique	Advantages	Limitations/future work	Accuracy (%)
1	Narayanana (32)	RF with SMOTE	High accuracy, sensitivity, and specificity in cardiac disease prediction using UCI heart dataset.	Focused only on the UCI dataset; more diverse datasets should be evaluated.	96.6
2	Ma et al. (20)	FlexiPulse sensor with ML	Accurate and affordable wearable tech for HD monitoring; uses ML for diagnostics	Needs large-scale validation for real-world implementation.	98.7
3	Kanagarathinam et al. (35)	Hybrid dataset with CatBoost	Achieved high accuracy using CV on the “Sathvi” dataset	Limited exploration of alternative classifiers and datasets.	98.11
4	Faizal et al. (22)	XG for heart failure prediction	Promising results for early mortality estimation and public health enhancement	Limited accuracy; opportunities exist to improve generalizability and robustness.	86.36
5	Li et al. (33)	Fusion models for CVD severity prediction	High accuracy in binary and multiclass classification, leveraging outputs from multiple algorithms.	Requires scalability and validation with larger datasets.	93–95 (binary); 72–75 (multiclass)
6	Proposed Optimized RST-ML Model	Integrating RST for feature selection with diverse ML classifiers and MCDM techniques.	Efficient feature selection with RST and enhanced accuracy through MCDM-based ranking (TOPSIS).	Needs validation on broader datasets and real-world clinical scenarios.	**98**.**70**

### 3.11 Proposed model validation with different open source datasets

The proposed Optimized RST-ML approach with MCDM-based ranking was evaluated across multiple open-source datasets to validate its efficiency in HD prediction. The datasets utilized for validation include the Obesity Levels dataset, Breast Cancer Wisconsin Diagnostic dataset, and CKD dataset. These datasets were chosen due to their varying characteristics, allowing for a comprehensive assessment of the proposed method's robustness.

The Obesity Levels dataset (56) comprises 2,111 records collected from Mexico, Peru, and Colombia. It includes 17 attributes related to eating habits and physical activities, classifying individuals into different obesity levels. A significant portion (77%) of this dataset was synthetically generated using the SMOTE technique, while the remaining 23% was collected directly from users via a web platform. The dataset provides a real-world scenario with categorical, binary, and continuous features, making it a challenging yet valuable resource for obesity classification.

The Breast Cancer Wisconsin Diagnostic dataset (57) contains 569 instances and 30 real-valued features extracted from digitized images of fine needle aspirates of breast masses. These features describe the characteristics of cell nuclei and are used to classify tumors as benign or malignant. The dataset does not contain missing values, making it suitable for evaluating the classification accuracy of various ML models.

The CKD dataset (58) consists of 400 records and 25 features, including critical health indicators such as red blood cell count, white blood cell count, and blood pressure levels. The target variable, “classification,” indicates whether a patient has CKD or not. The dataset required preprocessing due to missing values, and all rows containing NaNs were removed as per standard data cleaning protocols. This dataset is particularly relevant to the study due to its medical nature and direct implications for HD prediction. The performance of the proposed method was validated using these datasets by evaluating key performance metrics. The results are presented in the Table 7.

**Table 7:** Performance comparison of the proposed method on different datasets.

Parameters	Obesity levels	Breast cancer Wisconsin	Chronic kidney disease
*Ac*(%)	95.51	98.33	96.49
*Pr*	0.94	0.98	0.95
*Sn*	0.95	0.99	0.95
*F*1	0.94	0.98	0.95
MCC	0.93	0.97	0.92
MAE	0.09	0.03	0.03
*Tt* (s)	0.23	0.06	0.12

The proposed approach achieved outstanding performance. The Obesity Levels dataset, the model attained 95.51% accuracy, demonstrating its effectiveness in handling synthetic and real-world data. The Breast Cancer Wisconsin dataset yielded 98.33% accuracy, indicating the model's ability to distinguish between benign and malignant tumors. Finally, for the CKD dataset, the model achieved an accuracy of 96.49%, proving its capability in medical diagnostics. These results highlight the effectiveness of the proposed Optimized RST-ML approach in various domains, particularly in medical and health-related datasets, showcasing its potential for real-world applications in disease prediction and classification.

## 4 Discussion

The proposed work demonstrates the potential of a stacked ensemble model combined with RST for accurate and interpretable prediction of HD risk. Several important methodological choices and limitations are discussed below, alongside directions for future research.

The adoption of RST for feature selection over modern feature selection techniques due to its inherent ability to handle uncertainty and imprecision without requiring prior data assumptions or parameter tuning. Unlike statistical or embedded methods such as Lasso, RST identifies minimal attribute subsets (reducts) based on data dependencies, thereby preserving classification accuracy while offering high interpretability and transparency which is critical in healthcare applications. Compared to statistical or black-box techniques like Lasso or embedded models, RST provides a rule-based, explainable framework while preserving classification accuracy during dimensionality reduction. This makes RST, and Johnson's Algorithm in particular, a robust and suitable choice for the proposed Optimized RST-ML model.

To ensure the robustness of the ensemble learning framework, each base model in the best-performing stack, Stack 4, was independently optimized using GridSearchCV. However, due to computational constraints and the complexity of hierarchical model tuning, hyperparameter optimization was not extended to the other stacked ensembles. Fine-tuning the final stacked models could potentially enhance predictive performance and may also influence the outcomes of the MCDM-TOPSIS ranking procedure used for model selection. Future work will investigate this possibility to assess whether ensemble-level optimization yields improved or different performance rankings.

Although the current analysis is limited to clinical datasets, the growing importance of non-invasive and wearable sensor data in cardiovascular health monitoring is recognized. Real-time data streams from such technologies could significantly enrich early detection and personalized risk stratification. Future research will incorporate these emerging data sources to enhance the clinical relevance and dynamic applicability of the proposed framework.

Furthermore, Country-level validation could offer deeper insights into model generalisability, as differences in population health indicators, healthcare systems, and risk factor distributions across countries may affect performance. Due to the limited availability of complete records, such validation was not feasible in the proposed work. Future work will consider larger, more balanced international datasets to assess cross-regional robustness and identify potential biases in predictive accuracy.

In summary, this study lays the foundation for a scalable and explainable HD prediction model. By integrating RST-based feature selection, ensemble learning, and multi-criteria decision-making, the study provide a comprehensive and transparent approach that balances accuracy, interpretability, and clinical relevance. Future research will focus on enhancing generalisability, leveraging wearable health data, and refining ensemble tuning to further strengthen the utility of the proposed model in diverse healthcare settings.

## 5 Conclusion and future work

This study proposed a hybrid framework that integrates RST for feature selection, ensemble ML through stacking, and MCDM using the TOPSIS technique to enhance classification performance in HD prediction. By employing Johnson's algorithm, the model effectively reduced dimensionality by preserving critical features. Multiple ML classifiers were trained and evaluated, and their strengths were assessed through five stacking configurations (Stack-1 to Stack-5). Pearson correlation analysis was used to ensure synergy among base learners in each stack, minimizing redundancy and maximizing learning diversity.

To identify the best-performing ensemble, a structured decision-making strategy was employed using the TOPSIS method, supported by MEREC-based weighting for objective criteria evaluation. Among the evaluated stacks, Stack-4 comprising advanced tree-based classifiers achieved the highest performance. Stack-4 was fine-tuned using GridSearchCV, with XG emerging as the best-performing model, achieving an accuracy of 98.70% and 100% precision, thereby demonstrating its effectiveness in high-stakes medical diagnostic tasks. To ensure interpretability, XAI techniques were utilized to analyse feature contributions and model behaviour, offering transparent insights crucial for clinical decision-making.

The framework was validated not only on the HD dataset but also across diverse domains using publicly available datasets such as Obesity Levels, Breast Cancer Wisconsin, and CKD. The consistently high performance across all datasets demonstrates the model's generalizability and adaptability. This highlights the strength of integrating RST, ML, and MCDM into a single unified diagnostic framework, which is relatively unexplored in current medical AI applications.

Future research will focus on validating the framework using large-scale, real-world clinical data to assess its robustness and scalability. Furthermore, integrating non-invasive and wearable sensor data holds potential to enhance personalized monitoring and early detection. Lastly, although classical ML algorithms were used in this study, future work can explore DL integration with RST to improve feature learning and prediction performance in complex, high-dimensional datasets. Together, these directions aim to strengthen the clinical applicability and generalisability of the proposed model across diverse healthcare settings.

## Data Availability

The original contributions presented in the study are included in the article/Supplementary Material, further inquiries can be directed to the corresponding author.
